# Functional Haplotypes in the *ADIPOQ* Gene are Associated with Underweight, Immunosuppression and Viral Suppression in Kenyan HIV-1 Infected Antiretroviral Treatment Naive and Experienced Injection Substance Users

**DOI:** 10.4314/ejhs.v30i4.4

**Published:** 2020-07-01

**Authors:** Shaviya Nathan, Budambula Valentine, Were Tom

**Affiliations:** 1 Department of Medical Laboratory Sciences, Masinde Muliro University of Science and Technology, Kakamega, Kenya; 2 Department of Environmental Health, Technical University of Mombasa, Kenya

**Keywords:** ADIPOQ gene, Injection substance use, Haplotypes

## Abstract

**Background:**

Human immunodeficiency virus and injection substance use have an influence on genes and gene expression. These effects could be beneficial or detrimental in defining disease outcomes. Adiponectin gene is key in modulating metabolic and immunoregulatory functions. Understanding the effects of human immunodeficiency virus and injection substance use on the gene in the context of antiretroviral therapy is important for predicting disease outcomes.

**Methods:**

This cross-sectional genetic study determined polymorphisms in the promoter region of adiponectin gene. Two variants were analyzed: rs2241766 and rs266729. Polymorphisms were associated with clinical markers of disease outcome; underweight, immunosuppression and viral suppression. The variants were genotyped via random fragment length polymorphism.

**Result:**

GC haplotype was associated with higher odds of having underweight (OR, 2.21; 95% CI, 1.83–4.60; P=0.008 *vs.* OR, 2.30; 95% CI, 1.89–4.71; P=0.006) in antiretroviral treatment - naive and experienced injection substance users and immunosuppression (OR, 1.90; 95% CI 1.67–3.98, P=0.041) in naive. Bonferroni correction revealed GC haplotype carriers only to have low body mass index in both naive (median, 14.8; IQR, 3.2 kg/m^2^; P=0.002) and experienced (median, 15.2; IQR, 3.2 kg/m^2^; P=0.002) injection substance users. Circulating total adiponectin levels were higher in naive (median, 19.5; IQR, 7.9 µg/ml) than - experienced (median, 12.0; IQR, 4.4 µg/ml) injection substance users (P=0.0001). GC carriers presented with low serum adiponectin levels in both study groups.

**Conclusion:**

The study revealed haplotypes of adiponectin gene at loci rs2241766 and rs266729 that could determine disease outcomes in human immunodeficiency virus -1 antiretroviral treatment- naive and experienced injection substance users.

## Introduction

Of an estimated 13 million people who inject illicit drugs in the world, 1.7 million are HIV infected (1). In Africa, ~630,000 people are injection substance users (ISUs) of whom 13.6% are living with HIV (2). In Kenya, over 50,000 ISUs have been identified, and the number is on the increase predominantly in coastal, urban, peri-urban areas and cities (3). The prevalence of HIV infection in Kenyan ISUs is ~18.3% with higher incidence of 18.7% mainly at the coast linked to high rates of injection substance use (4).

Adiponectin gene (*ADIPOQ*) encodes adiponectin (5), a key adipokine regulating carbohydrate and fat metabolism, immune and inflammatory responses (6). *ADIPOQ* gene is ~15.8 kb spanning across three exons on chromosome 3q27 (7). *ADIPOQ* gene is highly expressed in adipocytes, and moderately in renal epithelial cells, macrophages and CD4+ T cells (8,9). Genetic variations in the *ADIPOQ* gene promoter loci -676 to +41 notably rs2241766, rs266729, rs17300539, rs16861210, rs6444175 and rs1501299 are the most extensively studied in relation to disease outcomes across populations (10). The rs2241766 variants are associated with metabolic syndrome, obesity and hypertension (10,11), while rs2241766 and rs266729 genotypes and haplotypes correlate with BMI in diabetics (12). Moreover, rs266729 is associated with nonalcoholic fatty liver disease in smokers (13), and onset of type-2 diabetes mellitus (14). In HIV infection, rs266729 GG variant is associated with low CD4+ T cell counts among patients on antiretroviral treatment (ART) (15). Also, a review of the two loci using ENCODE, haploreg, HapMap and regulome databases revealed rs2241766 to be a synonymous SNP with enhancer histone markers in blood while rs266729 as an intronic variant associated with *ADIPOQ* gene expression.

It appears that *ADIPOQ* variants mediate disease outcomes by controlling gene expression. For instance, *in vitro* studies in recombinant adiponectin treated HEK293 cells deficient in metabolic activity showed increased expression of the *ADIPOQ* gene in skeletal muscles (16). Moreover, several in vivo studies also showed that polymorphisms in the *ADIPOQ* gene influence gene expression which translates into altered circulating adiponectin levels (17). In addition, *ADIPOQ* TG haplotype at rs2241766 and rs266729 loci, and at +45 and +276 loci were associated with low circulating adiponectin levels in insulin resistant Korean and Indian patients, respectively (18). Altogether, these findings suggest that haplotypes are better predictors of circulating adiponectin levels and disease progression. Additionally, underweight, immunosuppression and viral suppression are key clinical markers of disease progression in both HIV-infected and non-infected ISUs (19). However, there is limited data on how *ADIPOQ* gene polymorphisms influence these markers of disease progression among HIV-1 ISUs. We have previously shown that serum adiponectin levels are low in HIV-1 ART-naive ISUs from coastal Kenya (20). In addition, injection substance use leads to epigenetic modifications in gene expression and causes alterations in the DNA sequences (17). Furthermore, HIV infection promotes a repertoire of up-regulations and/or down-regulations of human gene expression (21). Consequently, HIV-1 ISUs provide a unique population in which gene regulation can be studied. Therefore, we examined associations of *ADIPOQ* gene variants at rs2241766 and rs266729 loci with underweight, immunosuppression and viral suppression among HIV-1 ART naive and experienced Kenyan ISUs.

## METHODS

### Study site and population

This cross-sectional study was conducted among HIV-1 ART-naive and -experienced ISUs from Mombasa, a coastal city in Kenya. Detailed description of the study site and population, clinical and adiponectin measurements are presented in our recent publications (22). In the current study, the ISUs were stratified into ART-naive and -experienced individuals. ART-naive ISUs were individuals testing positive for HIV-1, reporting having never used ART and having no AIDS defining illnesses. ART-experienced were individuals testing positive for HIV-1, currently on ART and having no AIDS defining illnesses. The clinical measures of underweight, immunosuppression and viral suppression were used in examining *ADIPOQ* gene regulation of HIV-1 infection in ISUs. Underweight was defined by BMI =18.5 kg/m^2^ (23). Although WHO classifies HIV infection into four immunologic stages based on CD4+ T cell count (24), immunosuppression was based on CD4+ T cell count =500 cells/µl. Viral suppression was also defined as per the WHO using >1000 HIV RNA copies/ml (25).

### CD4 T cell enumeration

CD4+ T cell counts were determined in an automated fashion using the BD FACSCalibur flow cytometer (Becton-Dickinson™, Franklin Lakes, USA). Briefly, 5.0 µl of EDTA blood samples were placed in a tube and RBC lysis buffer added. After 5 minutes incubation, the cells were washed and fluorescent-tagged antibodies (anti-CD3, anti-CD4, and antiCD45) were added. The cells were incubated for 30 minutes after which the samples were washed and the CD4+ T cells enumerated on the flow cytometer.

### HIV-1 viral load

HIV-1 viral loads were determined using the automated Abbott m2000 System according to the manufacturer’s instructions (Abbott Molecular Inc., Illinois, USA). Briefly, RNA was extracted from 0.2 ml serum samples and reverse-transcribed into cDNA. The cDNA was amplified using HIV-1-specific and internal control primers. Fluorescence intensity of the HIV-1 probe was converted into viral loads by the analyzer

### Serum adiponectin measurement

Total adiponectin concentrations were determined in serum using commercial ELISA reagents according to the manufacturer's protocols (R&D Systems, Minneapolis, MN, USA).

### SNP selection strategy

SNPs rs2241766 and rs266729 were selected based on >5% distribution of alleles in HapMap populations (26,27). Also, location/co-localization with putative transcription factor binding sites and reported association with adiponectin levels was used. Furthermore, other adiponectin related traits and their closeness in genomic proximity were considered

### DNA extraction and genotyping

DNA was extracted from dry blood spot (Whatman plc) samples using QiaAmp™ DNA Mini Kit (Qiagen Inc., Valencia, USA) according to the manufacturer’s protocols. Genotyping was carried out by PCR-restriction fragment length polymorphism (RFLP). PCR of DNA segments, containing rs2241766 and rs266729, was performed using the following primers: rs2241766, forward primer 5'GCAGCTCCTAGAAGTAGACTCTGCTG -3' and reverse primer 5'-GCAGGTCTG TGATGAAAGAGGCC -3'; rs266729, forward primer 5'-GGTGGACTTGACTTTACTGG -3', and reverse primer 5'TAGAAGCAGCCTGGAGAA -3'. PCR was carried out in a final volume of 50 µL containing: 5 µL 10X PCR buffer (KEMTAQ®), 4 µL MgCl2, 2 µ dNTP, 10 pmol of each primer, 0.5 µL Taq DNA polymerase, 32.5 µL ddH2O, and 4 µL genomic DNA. The cycling conditions for rs2241766 and rs266729 were 94°C for 2 minutes, 35 cycles of 94°C for 15 seconds, 60°C for 30 seconds, and 72°C for 30 seconds. This was followed by a final extension at 72°C for 10 minutes and a pause at 4°C. The PCR products were separated on a 2% UltraPure™ agarose gel and visualized under UV light. PCR products were digested with 10 U/µL restriction enzymes (New England Biolabs, Ipswich, USA). SNPs rs2241766 (*SmaI*), and rs266729 (*HhaI*) were genotyped by PCR-RFLP. The digested products were separated on a 2% NuSieve® 3:1 agarose gel (NuSieve® GTG® Agarose). The digests on agarose gel were read and scored separately by two different trained technologists-in the case of disagreement a third trained research assistant was called in. Samples that did not amplify for both variants were removed during analysis.

### Data analysis

Statistical analysis was performed using Statistical Package for the Social Sciences (SPSS) version 24.0 (SPSS Inc, Chicago, USA) and GraphPad Prism version 6.0 (GraphPad, California, USA). Continuous data such as age, height, weight, BMI, CD4+ T cell counts, log10 HIV-1 RNA copies and adiponectin levels were compared between ART-naive and -experienced ISUs using the Mann-Whitney U test. Discrete data and categorical data were compared between the study groups using the Fisher’s exact tests. Hardy-Weinberg equilibrium was determined using the Online Encyclopedia for Genetic Epidemiology studies software at www.oege.org/software/hwe-mr-calc.shtml. Haplotype distribution was determined using the HPlus haplotype analysis software-http://cdsweb01.fhcrc.org/HPlus/ and Haploview-www.broadinstitute.org/haploview. Kruskal-Wallis test was used to determine acrosshaplotype differences, and where significant, post-hoc Mann-Whitney U tests with the Bonferroni correction were performed with cut-off set at P=0.002. Binary logistic regression analyses were used to determine the associations of haplotypes with underweight, immunosuppression and viral suppression. In these regression models, we controlled for age, gender, duration and frequency of drug injection in the ART-naive; and age, gender, duration and frequency of drug injection, and duration of ART use in the ART-experienced ISUs. All tests were two-tailed and P=0.05 was used for statistical inferences.

### Ethics approval and consent to participate

Ethical approval for this study was obtained from Kenyatta University Ethics Review Committee (Protocol: PKU019/116/2012) and Masinde Muliro University of Science and Technology Institutional Ethical Review Committee (Protocol: MMU/COR403012-V27). Written informed consent was obtained from each participant before enrolment. All HIV-1 infected ART-naive study participants were referred for further treatment and care at the HIV Comprehensive Care Clinics

## Results

### Demographic and clinical profiles of the study participants

The demographic and clinical characteristics of the study participants are summarized in Table 1. A total of 284 adults (males, *n*=118 and females, *n*=166) comprising of HIV-1 infected ISUs (ART-naive, *n*=108 and ART-experienced, *n*=176) were recruited into the study. Age (median, 30.7; IQR, 6.7 years *vs.* median, 30.2; IQR, 6.3 years; *P*=0.708), gender (females, 53.7% *vs.* males 61.4%) and body height (median, 1.7; IQR, 0.2 meters *vs.* median, 1.6; IQR, 0.4 meters; *P*=0.197) were comparable between the ART-naive and experienced subjects, respectively.

Median body weight was significantly lower in ART-experienced (median, 53.7; IQR, 7.1 kg) compared to ART-naive (median, 49.5; IQR, 4.7 kg) ISUs (*P*=0.007). Likewise, BMI was significantly lower in ART-experienced (median, 14.1; IQR, 2.2 kg/m^2^) relative to ART-naive (median, 17.7; IQR, 1.5 kg/m^2^) ISUs (*P*=0.021). Consistent with the lower BMI, the rate of underweight was higher in ART-experienced (47.2%) than in the ART-naive (31.5%) ISUs (*P*=0.009). HIV-1 RNA copies/ml were similar between ART-naive and -experienced ISUs (median; 2.6; IQR, 2.3 log_10_ HIV-1 RNA copies/ml *vs* median; 2.2; IQR, 2.1 log_10_ HIV-1 RNA copies/ml *P*=0.291) as were the rates of viral suppression (50.0% *vs.* 60.2%; ^P^=0.170), respectively. CD4+ T cell counts were also similar between the study groups (ARTnaive; median, 482; IQR, 434 cells/µl; -experienced; median, 408; IQR, 407 cells/µl; *P*=0.321). Consequently, the rates of immunosuppression were also similar among the groups (ART-naive, 50.0% and -experienced, 56.3%; *P*=0.109).

**Table 1:** Demographic and clinical profiles of the study participants

Characteristic	HIV-1[+] ART [-], n=108	HIV-1[+] ART [+], n=176	P
Age, yrs.	30.7(6.5)	30.2 (6.3)	0.708
Male/Female, n (%)	50/58(46.3/53.7)	68/108(38.6/61.4)	0.126
Height, m	1.7(0.2)	1.6(0.4)	0.197
Weight, kg	53.7(7.1)	49.5(4.7)	**0.007**
Log10HIV-1 RNA copies/ml	2.6(2.3)	2.2(2.1)	0.291
=1000	54(50.0)	106(60.2)	0.170
≥1000	54(50.0)	70(39.8)	
BMI, kg/m^2^	17.7(1.5)	14.1(2.2)	**0.021**
=18.5	34(31.5)	83(47.2)	**0.009**
≥18.5	74(68.5)	93(52.8)	
CD4+ T cells/µl	482(434)	408(407)	0.321
=500	54 (50.0)	99(56.3)	0.109
≥500	54(50.0)	77(43.7)	

### Haplotype frequencies

**Figure 1:**
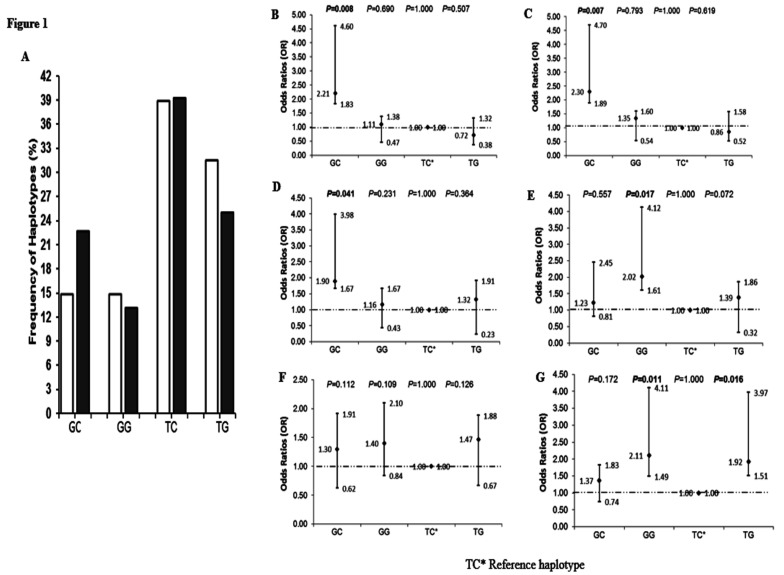
*ADIPOQ* haplotype distribution and relationship with underweight, immunosuppression and viral suppression in HIV-1 ART-naive and -experienced ISUs. (A) Open and closed bars represent frequency (%) of haplotypes in -naive and -experienced ISUs, respectively. (B) Haplotypes *vs* underweight among -naive. (C) Haplotypes *vs* underweight among -experienced. (D) Haplotypes *vs* immunosuppression among -naive. (E) Haplotypes *vs* immunosuppression among -experienced. (F) Haplotypes *vs* viral suppression among -naive. (G) Haplotypes *vs* viral suppression among -experienced. Values in bold are significant P-values at a cut-off of P=0.05.

Haplotype frequencies are shown in Figure 1A. The TC haplotype was the most prevalent, with about equal distribution in the ART-naive (38.9%) and experienced (39.2%) ISUs (*P*=0.418). While the GC haplotype frequency was higher in ART-experienced (22.7%) compared to -naive (14.8%) ISUs (*P*=0.031), the TG haplotype carriage was higher in the ART-naive (31.5%) compared to -experienced (25.0%) ISUs (P=0.007). GG haplotype distribution was similar in the study groups (naive, 14.8% *vs.* experienced, 13.1%; *P*=0.214), respectively. Haplotype frequency distribution was consistent with HWE in the -naive (χ2=0.52, *P*=0.471) and -experienced (χ2=0.42, *P*=0.517).

### Haplotype distribution in HIV-1 clinical outcomes

Haplotype distribution in different HIV-1 clinical outcomes is shown in Table 2. TC haplotype was the reference haplotype. Relative to TC, GC haplotype carriage was higher in underweight ART-naive (68.8% *vs* 31.2%; *P*=0.001) and -experienced (62.5% *vs* 37.5%; *P*=0.002) ISUs. Likewise, most of the GC haplotype carriers were immunosuppressed in both -naive (62.5% *vs* 37.5%; *P*=0.002) and experienced (67.5% *vs* 32.5%; *P*=0.001) ISUs. GC haplotype carriers in both -naive (68.8% *vs* 31.2%; *P*=0.001) and -experienced ISUs (62.5% *vs* 37.5%; P=0.002) were more virally suppressed.

**Table 2:** Haplotype distribution in HIV-1 disease outcomes

Haplotype	HIV-1[+] ART [-], n=108									HIV-1[+] ART [+], n=176								
	BMI			CD4 count			Viral load			BMI			CD4 count			Viral load		
	=18.5	≥18.5	*P*	=500	≥500	*P*	=1000	≥1000	*P*	=18.5	≥18.5	*P*	=500	≥500	*P*	=1000	≥1000	*P*
GC	11	5	**0.001**	10	6	**0.002**	11	5	**0.001**	25	15	**0.002**	27	13	**0.001**	25	15	**0.002**
GG	8	8	0.998	9	7	0.178	7	9	0.441	11	22	0.763	22	11	0.763	23	10	0.132
TC*	15	27	1.000	13	29	1.000	26	16	1.000	27	42	1.000	25	44	1.000	44	25	1.000
TG	17	17	0.998	19	15	0.248	18	16	0.700	23	21	0.546	23	21	0.451	22	22	0.998

Data are presented as numbers (n) of subjects. HIV-1, human immunodeficiency virus type-1. ART [-], antiretroviral treatment-naive. ART [+], anti-retroviral treatment-experienced. Data analysis was conducted using chi-square tests. Values in bold are significant *P*-values. TC*-reference haplotype.

### Variation in HIV-1 clinical outcomes across haplotypes

Variation of clinical outcomes across haplotypes is shown in Table 3. BMI was significantly different across haplotypes in both naive (*P*=0.002) and experienced (*P*=0.002) ISUs. CD4 T cell counts only differed across haplotypes in naive (*P*=0.002) ISUs. Post-hoc correction analyses revealed that the GC (median, 14.8; IQR, 3.8 kg/m^2^ *vs* 15.2; IQR, 3.2 kg/m^2^) haplotype carriers had significantly lower BMI compared to GG (median, 18.7; IQR, 1.4 kg/m^2^ *vs* 18.3; IQR, 1.7 kg/m^2^), TC (median, 18.7; IQR, 2.2 kg/m^2^ *vs* 18.5; IQR, 2.4 kg/m^2^) and TG (median, 20.1; IQR, 3.9 kg/m^2^ *vs* 18.8; IQR, 1.5 kg/m^2^) in both naive and experienced ISUs (P=0.002), respectively. In contrast, the CD4 T cell counts were higher in TC (median, 617; IQR, 314.5 cells/µl) haplotype carriers relative to GC (median, 461; IQR, 262.8 cells/µl), GG (median, 442; IQR, 194 cells/µl) and TG (median, 464; IQR, 293.3 cells/µl) haplotype carriers in both naive ISUs only (*P*=0.002), respectively.

**Table 3:** Variation of HIV-1 disease outcomes across haplotypes

Characteristic	HIV-1[+] ART [-], n=108					HIV-1[+] ART [+], n=176				
	GC, n=16	GG, n=16	TC, n=42	TG, n=34	P	GC, n=40	GG, n=23	TC, n=69	TG, n=44	P
Underweight	14.8 (3.8)	18.7 (1.4)^a^	18.7 (2.2)^b^	20.1 (3.9)^c^	**=0.0001^a,b,c^**	15.2 (3.2)	18.3 (1.7)^a^	18.5 (2.4)^b^	18.8 (1.5)^c^	=0.0001^a,b,c^
Immunosuppression	461.0 (262.8)	442.0 (194)	617.0 (314.5)^b,d^	464.0 (293.3)^e^	=0.0001^b,d,e^	364.0 (328)	694.0 (917.0)^a^	876.0 (563.0)^b^	523.0 (437.8)^c,d^	**0.021^a,b,c,d^**
Viral suppression	2.5 (2.6)	4.9 (3.6)^a^	2.2 (2.0)^d^	2.5 (1.8)^e^	0.064	2.7 (0.7)	3.9 (2.6)	2.6 (1.8)	2.5 (2.7)	0.135

Data are presented as medians (IQR). Bonferroni correction set at *P*=0.002. Significant comparisons between haplotypes are indicated as; ^a^*P*=0.002 *vs* GG, TC and TG, ^b^*P*=0.002 *vs* GC, GG and TG.

### Association of haplotypes with underweight

Previous studies showing that underweight is linked to low serum adiponectin levels and predicts wasting in HIV infection and substance use (28), led us to examine the relationship of *ADIPOQ* haplotypes and underweight. Associations of *ADIPOQ* haplotypes with underweight are shown in Figure 1B and C. The GC haplotype was associated with higher probability of having underweight in both ART-naive (OR, 2.21; 95% CI, 1.83–4.60; *P*=0.008) and -experienced (OR, 2.30; 95% CI, 1.89–4.71; *P*=0.006) ISUs.

### Association of haplotypes and immune suppression

Since adiponectin promotes CD4+ T cell depletion and decreased CD4+ T cell numbers predicts progression of HIV infection (29), we examined the association between *ADIPOQ* haplotypes and immunosuppression. Associations of *ADIPOQ* haplotypes with immune suppression are shown in Figure 1D and E. This analysis revealed that GC (OR, 1.90; 95% CI 1.67–3.98, *P*=0.041) and GG (OR, 2.02; 95% CI 1.61–4.12, *P*=0.017) haplotypes were associated with a higher probability of having immunosuppression in ART-naive and -experienced ISUs, respectively.

### Association of haplotypes with viral suppression

The viral load is associated with serum adiponectin and progression of HIV infection (30); hence, we examined whether *ADIPOQ* haplotypes were associated with viral suppression. Associations of *ADIPOQ* haplotypes with viral suppression are shown in Figure 1F and G. Analysis of the haplotypes revealed that GG (OR, 2.11; 95% CI 1.49–4.11, *P*=0.011) and TG (OR, 1.92; 95% CI 1.51–3.97, *P*=0.016) haplotypes were associated with a higher probability of having viral suppression in ART-experienced ISUs only.

### Circulating adiponectin levels *vs.* clinical markers

**Figure 2:**
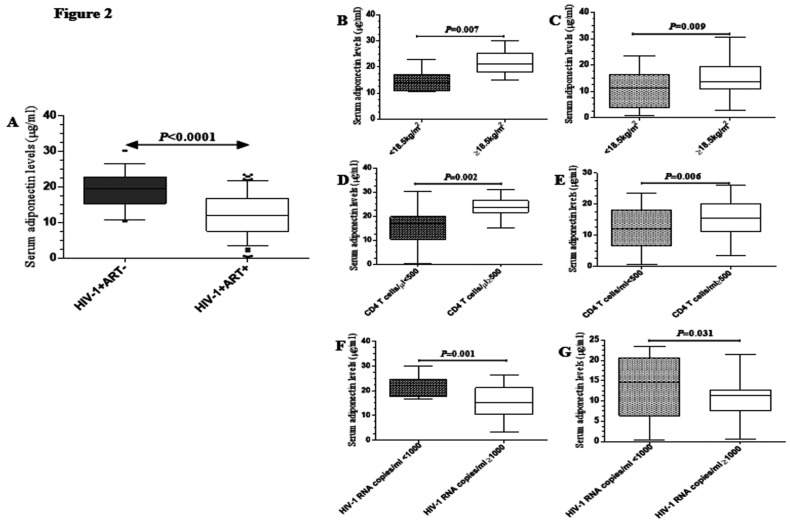
Serum adiponectin levels (µg/ml) in ART-naive and –experienced ISUs. (A) Overall serum adiponectin levels. Adiponectin levels by underweight, immunosuppression and viral suppression status in HIV-1 ISUs. Box plots showing adiponectin levels by underweight, immunosuppression and viral suppression status in -naive (B, D and F) and -experienced (C, E, and G)

ELISA measurement of circulating total adiponectin levels in serum specimens showed that ART-experienced (median, 12.0; IQR, 4.4 µg/ml) ISUs had higher serum adiponectin in comparison to ART-naive (median, 19.5; IQR, 7.9 µg/ml; *P*=0.0001; Figure 2A) ISUs. Subsequent analyses by clinical status indicated that adiponectin levels were reduced in individuals presenting with underweight among ART-naive (median, 13.9; IQR, 3.7 µg/ml *vs.* median, 21.3; IQR, 9.2 µg/ml; P=0.007; Figure 2B) and -experienced (median, 12.5; IQR, 2.2 µg/ml *vs.* median, 16.7; IQR, 4.2 µg/ml; *P*=0.009; Figure 2C) ISUs. Likewise, adiponectin levels were lower in individuals having immunosuppression in ART-naive (median, 19.5; IQR, 3.2 µg/ml *vs.* median, 26.9; IQR, 7.0 µg/ml; *P*=0.002; Figure 2D) and -experienced (median, 11.9; IQR, 1.3 µg/ml *vs.* median, 13.2; IQR, 3.8 µg/ml; *P*=0.006; Figure 2E) ISUs. Moreover, individuals with viral suppression among ART-naive (median, 19.9; IQR, 6.4 µg/ml *vs.* median, 15.2; IQR, 4.7 µg/ml; *P*=0.001; Figure 2F) and experienced (median, 16.5; IQR, 5.1 µg/ml *vs.* median, 11.1; IQR, 2.5 µg/ml; *P*=0.031; Figure 2G) had lower adiponectin levels.

### Association of haplotypes with circulating adiponectin levels

**Figure 3:**
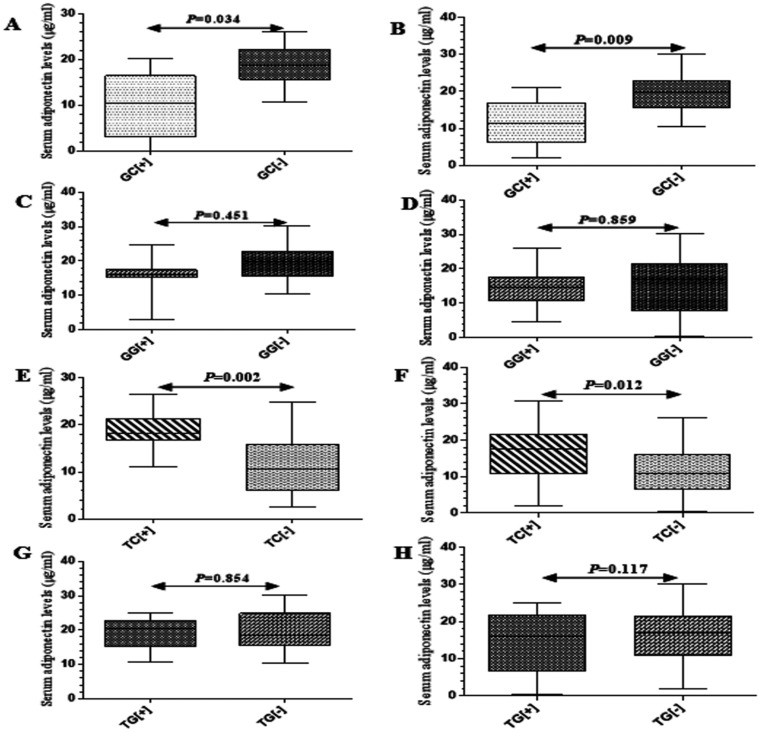
Box plots showing serum adiponectin levels in GC, GG, TC and TG haplotype carriers among -naive (A, C, E and G) and -experienced (B, D, F and H) ISUs.

Since all the clinical markers of HIV infection were associated with decreased circulating adiponectin levels, we determined associations between* ADIPOQ* haplotypes and adiponectin levels (Figure 3). Carriers of the GC haplotype had lower adiponectin levels compared to non-carriers in both ART-naive (median, 17.3; IQR, 5.3 µg/ml *versus* median, 28.2; IQR, 9.2 µg/ml; *P*=0.034; Figure 3A) and -experienced (median, 11.9; IQR, 5.7 µg/ml *vs.* median, 17.3; IQR, 3.7 µg/ml; *P*=0.009; Figure 3B) individuals. In contrast, adiponectin levels were higher in TC carriers than in non-carriers in ART-naive (median, 21.3; IQR, 2.9 µg/ml *vs.* median, 11.8; IQR 1.8 µg/ml; *P*=0.002; Figure 3E) and -experienced (median, 12.7; IQR, 5.8 µg/ml *vs.* median, 8.4; IQR, 4.6 µg/ml; *P*=0.012; Figure 3F).

### Discussion

This study focused on two variants of the *ADIPOQ* gene: rs2241766 and rs266729. Both variants occur in regions where transcription factors bind to the gene (11). We compared haplotype distribution between the two study groups. Four possible haplotypes were generated- GC, GG, TC, and TG. Rate of GC carriage was lower in the -naive compared to -experienced ISUs. In contrast, TG carriers were more in HIV-1 ART-naive ISUs compared with -experienced suggesting that *ADIPOQ* haplotypes could be influencing disease outcomes in HIV-1 ISUs. Previous studies have reported *ADIPOQ* haplotypes modulating obesity and type 2 diabetes (31). There is limited data on *ADIPOQ* haplotype modulation of disease outcomes in infectious diseases including HIV-1. This study is the first one to describe *ADIPOQ* haplotypes in HIV-1 infected ISUs.

Consistent with our previous study (20), ART-naive ISUs had higher serum adiponectin levels suggesting that ART could directly or indirectly be causing lower levels of circulating adiponectin. Previous studies have associated ART use with low levels of serum adiponectin in HIV infected individuals. For instance, stavudine has been shown to lower serum adiponectin up to 3.8 folds (32). In addition, a decrease in circulating adiponectin concentration has been reported in individuals on HAART (33). NRTIs have also been found to modulate lipodystrophy in HIV patients which is associated with altered circulating adiponectin levels (34). Evidence from genome wide association studies has revealed derangements that arise in genes involved in adipocyte differentiation and lipid metabolism on exposure to ARVs (35). Contrastingly, Morimoto et al., showed that hypoadiponectinemia exhibited by HIV-1 infected patients is not influenced by ART use (36). Taken together, these studies suggest a direct or an indirect effect of ART on the circulating levels of adiponectin.

Consistent with lower BMI, the GC haplotype was associated with higher odds of having underweight in both ART-naive and -experienced ISUs. The GC haplotype carriers also had a higher chance of having immunosuppression in both the ART-naive and –experienced individuals. These findings suggest that *ADIPOQ* haplotypes modulate HIV-1 infection and treatment outcomes. Indeed, *ADIPOQ* haplotypes have been previously associated with disease progression in several pathological conditions. For instance, the TG haplotype has been linked with disease outcomes for patients with insulin resistance, coronary heart disease and obesity (18). However, very few studies have investigated *ADIPOQ* haplotypes in the context of infectious diseases. Kato *et al* reported *ADIPOQ* haplotypes to be modulating serum adiponectin levels in HIV infected individuals (15), but no study has yet described *ADIPOQ* haplotypes in the context of HIV-1 ISUs. Our findings therefore imply that the GC haplotype could be driving deleterious disease outcomes in HIV-1 infected ISUs. Nonetheless, the association of *ADIPOQ* haplotypes with BMI in the current study should be interpreted with caution as age and gender of an individual correlate with BMI such that younger individuals and females are more likely to have a lower BMI (37).

Overall, our findings appear to predict that HIV-1, injection substance use, antiretroviral treatment and polymorphisms in the promoter of *ADIPOQ* gene modulate the circulating adiponectin levels. Furthermore, variations in the promoter region of the *ADIPOQ* gene may affect the affinity, avidity and magnitude for which the gene binds with its transcription factors both *cis* and *trans* upstream so as to initiate transcription. Therefore, this modulates transcription and subsequently translation altering the circulating adiponectin levels. This in turn affects disease outcomes in HIV-1 infected ART-naïve and - experienced ISUs.

In conclusion, this study revealed associations between *ADIPOQ* gene variant haplotypes and clinical markers in HIV-1 ISUs. *ADIPOQ* polymorphisms can thus be used as surrogate markers in HIV-1 infected ISUs.
